# Gut Microbiome Toxicity: Connecting the Environment and Gut Microbiome-Associated Diseases

**DOI:** 10.3390/toxics8010019

**Published:** 2020-03-12

**Authors:** Pengcheng Tu, Liang Chi, Wanda Bodnar, Zhenfa Zhang, Bei Gao, Xiaoming Bian, Jill Stewart, Rebecca Fry, Kun Lu

**Affiliations:** Department of Environmental Sciences and Engineering, University of North Carolina at Chapel Hill, Chapel Hill, NC 27599, USA; ptu@live.unc.edu (P.T.); liang16@live.unc.edu (L.C.); wanda_bodnar@unc.edu (W.B.); zhenfaz@email.unc.edu (Z.Z.); wintergb2012@gmail.com (B.G.); bxmroly@uga.edu (X.B.); jill.stewart@unc.edu (J.S.); rfry@unc.edu (R.F.)

**Keywords:** gut microbiome, environment, chemical toxicity

## Abstract

The human gut microbiome can be easily disturbed upon exposure to a range of toxic environmental agents. Environmentally induced perturbation in the gut microbiome is strongly associated with human disease risk. Functional gut microbiome alterations that may adversely influence human health is an increasingly appreciated mechanism by which environmental chemicals exert their toxic effects. In this review, we define the functional damage driven by environmental exposure in the gut microbiome as gut microbiome toxicity. The establishment of gut microbiome toxicity links the toxic effects of various environmental agents and microbiota-associated diseases, calling for more comprehensive toxicity evaluation with extended consideration of gut microbiome toxicity.

## 1. Introduction

The human gut microbiome including the microorganisms, their genomes, and the surrounding environment in the gut, has received unprecedented attention over the past decade [1]. Mounting evidence suggests that the metabolic activities in the gut microbiome are profoundly intertwined with human health and disease [2]. A number of important functions performed by the gut microbiome are well recognized including the digestion of polysaccharides, biosynthesis of vitamins and nutrients, colonization resistance, and immune system modulation [3,4,5]. Moreover, the effects of the gut microbiome on host metabolism and physiology extend beyond the gut to distant organs such as the liver, muscle, and brain [2,6]. Due to its crucial role in human fitness, the gut microbiome is now considered as a new organ in the human body [7,8,9]. It is unequivocal that the gut microbiome functions properly on the premise that a normal gut microbial homeostasis is maintained [2]. However, the constitution and functionality of the gut microbiome can be readily influenced by diverse intrinsic and extrinsic factors [10]. For example, exposure to various xenobiotics leads to functional perturbation in the gut microbiome [11,12,13,14,15]. Mounting studies suggest that these environmentally induced perturbations are potentially linked to elevated disease risks [2,16]. Adverse health outcomes including inflammatory bowel disease (IBD), obesity, diabetes, cardiovascular disease, liver disease, colorectal cancer, and neurological disorders can be at least in part attributed to undesirable functional alterations in the gut microbiome [17,18,19,20,21,22,23].

**Figure 1:**
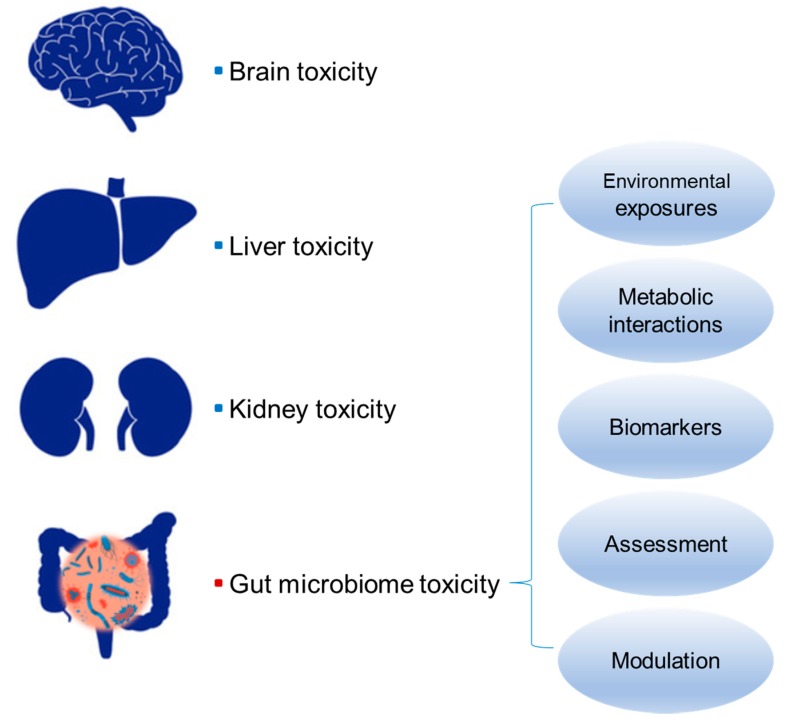
A potential new member of the organ toxicity family: gut microbiome toxicity. Toxicity of organs including brain, liver, and kidney is well defined and acknowledged. Similarly, the discussion of gut microbiome toxicity encompasses environmental exposures (causes), interactions between the gut microbiome toxicity and human diseases (mechanisms of gut microbiota-related diseases), biomarker and assessment (diagnosis), and modulation (treatment).

Certain environmental toxins can induce damage and dysfunction in the liver, which is termed as liver toxicity. Exposure to these toxins changes the morphology and functionality of the liver, hence leading to liver diseases. Similarly, exposure to some environmental chemicals causes structural differences and functional alterations in the gut microbiome, which probably results in a series of adverse health outcomes. For instance, arsenic exposure can perturb the composition and metabolites of the mouse gut microbiome, which potentially contributes to its toxicity [12]. Given the increasingly recognized role of the gut microbiome in human health coupled with its susceptibility to environmental insults, it is of significance to define gut microbiome toxicity. As the gut microbiome is viewed as a new human organ by its importance, we accordingly define the environmentally driven functional damage in the gut microbiome as gut microbiome toxicity. The development of gut microbiota-related diseases can be generalized as environmental factors leading to deleterious alterations in the gut microbiome, which adversely affect human health via host–gut microbiota interactions. In other words, gut microbiome toxicity triggered by environmental exposures contributes to gut microbiota-related adverse outcomes. By including gut microbiome toxicity into the organ toxicity family, we can now discuss the relationship between the environment and gut microbiota-related diseases in the context of toxicology (Figure 1).

Environmental exposure is a significant risk factor for a series of human diseases, overlapping those diseases that are associated with the gut microbiome [24,25,26]. Thus, gut microbiome toxicity may be the missing link between environmental exposure and microbiome-related human diseases. Moreover, the current toxicity testing system does not include toxic endpoints regarding the effects of environmental chemicals on the gut microbiome [27,28]. Considering the potential involvement of the gut microbiome in human disease, it is imperative to integrate gut microbiome toxicity into the toxicity assessment of environmental exposure. Thus, the establishment of gut microbiome toxicity may offer insights regarding the mechanistic basis underlying the toxicity of environmental chemicals, calling for more comprehensive risk assessment with the integration of gut microbiome toxicity. Additionally, environmentally driven alterations in the gut microbiome are not necessarily always adverse. By functional damage, we refer in particular to those that potentially contribute to adverse health outcomes, for instance, the production of pro-inflammatory metabolites. With the introduction of ‘gut microbiome toxicity’, we highlight the underappreciated mechanisms by which environmental factors lead to or exacerbate diseases through perturbing the gut microbiome functions.

In this review, we carefully define gut microbiome toxicity as environmentally driven functional damage in the gut microbiome. Functional damage may include changes in bacterial metabolites, loss of bacterial diversity, or effects on energy metabolism and balance. We focus on recent studies in support of the establishment of gut microbiome toxicity, and we accordingly discuss the environmental exposures, metabolic interactions in human disease, biomarkers and assessment, and modulation (Figure 1). Specifically, we review recent studies demonstrating the functional perturbation in the gut microbiome driven by various xenobiotics such as antibiotics, heavy metals, pesticides, and artificial sweeteners. These functional changes include, but are not limited to, alterations in the bacterial production of metabolites, diversity loss in the bacterial community, and interference in energy metabolism, which are further linked to the development of gut microbiota-related diseases. Moreover, microbiome changes including compositional and functional changes can serve as biomarkers for gut microbiome toxicity. Additionally, we briefly summarize current approaches for the assessment of gut microbiome toxicity as well as effective gut microbiome modulation.

## 2. Environmental Exposures

The fact that a number of xenobiotics can trigger gut microbiome toxicity suggests the underestimation of the toxic effects of specific chemicals. On one hand, the induction of gut microbiome toxicity may be considered a potential new mechanism by which known toxic chemicals (e.g., heavy metals, pesticides) lead to or exacerbate human diseases. On the other hand, it is of necessity to reconsider the health effects and acceptable daily intake (ADI) of widely-used chemicals such as food additives in the context of their contribution to gut microbiome toxicity. The impact of xenobiotics on the human gut microbiome can be direct or indirect. The human gut microbiome encodes more diverse metabolic enzymes, which greatly expands the repertoire of biochemical reactions within the human body [29]. Some environmental chemicals can directly affect the gut bacteria by interrupting specific metabolic pathway or gene expression, leading to distinct selection pressures, hence shaping the gut microbial community due to the uniqueness of the set of metabolic pathways and genome possessed by different bacterial species [29]. Therefore, the selection of resistant bacteria upon certain exposure could lead to an unbalanced gut eco-system. Additionally, some environmental chemicals can indirectly impact the gut microbiome through the influence on host physiology (e.g., gut mucosa [30]) and cell-to-cell communications of bacteria (e.g., quorum sensing [31]). That being said, the mechanistic basis underlying the microbial perturbation induced by specific chemical exposure remains elusive. Here, we highlight representative xenobiotics such as antibiotics, heavy metals, pesticides, and artificial sweeteners that cause gut microbiome toxicity with significant functional alterations.

### 2.1. Antibiotics/Drugs

It is commonly accepted that antibiotic administration, especially broad-spectrum antibiotics, severely impacts commensal bacteria. Both short-term and long-term antibiotic treatments lead to gut microbiome toxicity, although partial recovery may occur [32,33]. In many cases, effects of antibiotics on bacterial communities result in diversity loss and compositional imbalance [34]. Moreover, antibiotics not only disturb the gut microbiome at the compositional level, but also substantially change its functional profiles. For example, a recent study used a multi-omics approach to resolve the changes induced by beta-lactam in human gut microbiome [35]. The results showed that beta-lactam treatment caused both taxonomic and functional alterations in gut microbiome supported by alterations at the metagenomic, metatranscriptomic, metametabolomic, and metaproteomic levels. Antibiotic exposure in mice has been linked to diseases such as obesity and diabetes [36,37]. Aside from antibiotics, non-antibiotic drugs also affect the gut microbiome [38]. For instance, metformin [39], non-steroidal anti-inflammatory drugs [40], proton pump inhibitors [41], and atypical antipsychotics [42] are reported to have effects on the gut microbiome, although the health consequences remain underexplored. A most recent study tested 1200 marketed drugs by in vitro screening to investigate their effects on the gut microbiome [43]. A quarter of human-targeted drugs were discovered to have effects on the gut bacteria to some degree, indicating the potential of medication to induce gut microbiome toxicity.

### 2.2. Heavy Metals

Heavy metals continue to be a class of intensely-studied environmental contaminants. However, the role of heavy metals in gut microbiome toxicity still remains underappreciated. In fact, the gut bacteria play an important role in the biotransformation of heavy metals, which may promote or attenuate their toxicity. For example, human gut bacteria are able to transform inorganic arsenic into less toxic organic arsenic species [44], and demethylation of methyl-mercury by gut bacteria can generate more toxic inorganic mercury [45]. Rats exposed to heavy metals including arsenic, cadmium, cobalt, chromium, and nickel exhibited significant changes in their gut microbial compositions [46]. Moreover, the functional profiles in the gut microbiome can be perturbed by heavy metals. Four weeks of arsenic exposure in drinking water (10 ppm) caused significantly different metabolite profiles in the mouse gut microbiome [12]. Likewise, 13 weeks of arsenic exposure with an environment-relevant dose (100 ppb) also perturbed diverse bacterial metabolic pathways [47]. Arsenic-induced gut microbiome toxicity provided a new angle to look at the mechanism of arsenic toxicity. Follow-up studies evaluating arsenic metabolism further demonstrated that arsenic-induced gut microbiome toxicity can be affected by factors including host genetics [48], gender [49], and bacterial infection [50]. Moreover, different arsenic doses (10 ppm and 100 ppb) induced different levels of perturbation in the mouse gut microbiome, indicating the dose-dependent effects of arsenic exposure, which together with toxicity response thresholds of arsenic-induced gut microbiome toxicity need to be further defined. In addition, exposure to manganese and lead disturbs the gut microbial functions of mice with perturbed pathways and metabolites [51,52].

### 2.3. Pesticides

Excessive use of pesticides in agriculture has raised concern about their health effects. The argument that certain pesticides are safe to humans because their targeted pathways do not even exist in the human body fails to consider the microbes in the gut [53]. For example, herbicides like 2,4-dichlorophenoxyacetic acid (2,4-D), which impact plant hormones, may affect gut bacteria because not only plants but also bacteria can synthesize plant hormones [54]. Likewise, the shikimate pathway, the target of herbicide glyphosate, is commonly present in human gut bacteria [55,56]. In bacteria, this pathway has an important function linking the metabolism of carbohydrates to the biosynthesis of folates and aromatic amino acids. Several studies have demonstrated the association between gut microbiome toxicity and pesticide exposure. For example, the fungicide imazalil changed the composition of gut microbiome in zebrafish and mice [57,58]. Of interest, a recent study showed that the mouse gut microbiome was perturbed by 13 weeks of diazinon exposure (4 ppm) [13]. Bacterial genes and metabolites involved in neurotransmitter synthesis were significantly perturbed, suggesting that diazinon-induced gut microbiome toxicity with altered bacterial biosynthesis of the neurotransmitter may be partially responsible for the neurotoxicity of diazinon [59,60]. In addition, exposure to diazinon and malathion impacts the quorum sensing of gut bacteria, providing evidence that affecting bacterial communications may be one of the underlying mechanisms of gut microbial perturbations [61,62].

### 2.4. Artificial Sweeteners

Food additives have facilitated the development of the modern food industry. Normally, food additives (e.g., artificial sweeteners, emulsifiers, preservatives) are added in food products with an approved safe amount. Nevertheless, gut microbiome toxicity was not taken into consideration when the related standards were determined. Many artificial sweeteners are considered safe because they are poorly metabolized by the human body [29]. However, the gut bacteria are actively involved in the biotransformation. For example, cyclamate, which is currently banned in the USA, can be metabolized by gut bacteria into cyclohexylamine, which is carcinogenic [63]. Artificial sweeteners stevioside and xylitol can also be metabolized by the gut bacteria [64,65]. Several studies have demonstrated that some artificial sweeteners and emulsifiers were able to induce gut microbiome toxicity with potential gut microbiota-related health consequences. For instance, in an elegantly conducted study by Suez and colleagues, consumption of saccharin induced both compositional and functional changes in mouse gut microbiome that might be involved in the development of glucose intolerance [66]. Another study reported similar results with increased inflammatory levels in addition to gut microbial perturbation induced by saccharin in mice [15]. Additionally, artificial sweeteners acesulfame potassium [61], sucralose [67], aspartame [68], and neotame [69] can also perturb bacterial metabolites in concert with health implications including obesity and inflammation. Moreover, another study found that two commonly used emulsifiers altered mouse gut microbial composition together with elevated inflammatory levels [70].

### 2.5. Others

The above discussion is not intended to be exhaustive. More information could be referred to in recent reviews regarding the relationship between xenobiotics and the gut microbiome [11,29,71]. We emphasize functional changes in the gut microbiome induced by environmental exposure in the current review, therefore studies were included documenting not only the compositional shifts after exposure, but also functional alterations manifested by functional metagenomics and metabolomics. A rapidly-increasing list of xenobiotics is linked to gut microbiome toxicity. Some are commonly present in our daily life; a typical example is the antibacterial and antifungal agent triclosan. It has been repeatedly reported that triclosan induced changes in the gut microbiome using multiple animal models [14,72,73,74]. However, the effects of triclosan on human gut microbiome remain controversial [75]. Furthermore, exposure to nicotine (a major toxic component of tobacco smoke) also perturbed the gut microbiome, affecting bacterial production of neurotransmitters in mice [76]. Such a large range of chemicals that may induce gut microbiome toxicity supports the necessity of considering gut microbiome toxicity regarding the toxicity evaluation of environmental agents.

## 3. Relationship between Gut Microbiome Toxicity and Human Diseases

The mutually beneficial relationship between the gut microbiome and the host is built on the premise that a well-balanced gut microbiota is maintained [2]. However, when afflicted with gut microbiome toxicity, functional alterations occur in the gut microbiome. Although it is difficult to disentangle these alterations, changes in microbial metabolites, diversity loss, and interference in energy metabolism are three major types of microbial disturbances that may adversely impact the host health via multiple host–microbiota axes, potentially leading to increased disease risks. Therefore, gut microbiome toxicity is a new link between the environment and human diseases. It should be noted that not all changes in the gut microbiome associated with environmental exposure are necessarily adverse. Nevertheless, it is of significance to establish the role of the gut microbiome in the toxicity of a number of environmental toxic agents, which has been largely underappreciated in the chemical research of toxicity. In this part, we discuss the connection between gut microbiome toxicity and human diseases, providing some mechanistic insights regarding environmentally driven gut microbiome-associated diseases.

### 3.1. Changes in Microbial Metabolites

Production of functional metabolites by bacteria plays a key role in human health and disease [4]. Gut microbiome toxicity has an altered bacterial metabolite profile, which influences host metabolism and physiology in a significant way. First, numerous bacterial metabolites act as signaling molecules through binding to receptors and activating diverse signaling cascades. Pathogen-associated molecular patterns (PAMPs) including lipopolysaccharide (LPS) and peptidoglycan can bind to Toll-like receptor 4 and nucleotide-binding oligomerization domain, respectively; both of which lead to pro-inflammatory effects [77,78,79]. Classic metabolites of gut bacteria, short-chain fatty acids (SCFAs), and bile acids can also function as signaling molecules and bind to cellular receptors. Specifically, SCFAs can bind to G-protein-coupled receptors (GPCRs), and bile acids can bind to GPCR TGR5 and nuclear receptor farnesoid X receptor (FXR) [80]. Activation of signaling pathways is implicated in important biological functions; the gut microbiome may therefore contribute to human health and disease by regulating metabolic activities involved in the production of SCFAs and bile acids. For instance, SCFAs and bile acids can modulate the secretion of glucagon-like peptide-1 (GLP-1) by binding to GPR43 [81] and TGR5 [82], respectively, which affects insulin secretion and glucose homeostasis. Perturbation in those bacterial activities may affect the risk of diabetes. In addition, tryptophan metabolites produced by bacteria such as indole 3-propionic acid and indole-3-acetic acid regulate intestinal immune cells and barrier functions through the activation of aryl hydrocarbon receptor (AHR) and the pregnane X receptor (PXR) [83,84,85]. AHR activation is involved in inflammatory bowel disease (IBD) among other diseases, and it is suggested that a reduction in bacterial tryptophan metabolism may contribute to IBD [85]. Second, some bacterial metabolites are strongly associated with specific diseases and phenotypes. A compelling example is the association of trimethylamine N-oxide (TMAO) and cardiovascular disease [19]. The gut bacteria can convert dietary components choline and L-carnitine to trimethylamine (TMA), which is further metabolized into TMAO in the liver. Gut microbiome-derived TMAO is highly correlated with cardiovascular disease risks. Likewise, products of protein fermentation (e.g., N-nitroso compounds, polyamines) derived by gut bacteria exert carcinogenetic effects and promote colorectal cancer [21]. Third, microbiome-derived metabolites play a role in brain functions through the gut–brain axis, many of which are neurotransmitters or their precursors (e.g., serotonin, gamma-aminobutyric acid) [4]. As mentioned previously, bacterial metabolites that are neurotransmitters were perturbed by environmental chemicals such as organophosphate diazinon and nicotine, which may partially explain their neurotoxicity. Additionally, the gut microbiome is an important source of beneficial vitamins and nutrients, therefore reduction in the bacterial production of those beneficial metabolites could be detrimental to human health [86]. Taken together, these examples support that gut microbiome toxicity can lead to diseases via altered metabolite profiles.

### 3.2. Diversity Loss

Diversity loss has been associated with many microbiota-related diseases such as IBD [87,88], irritable bowel syndrome (IBS) [89], acute diarrhea [90], and *Clostridium difficile*-associated disease (CDAD) [91]. Trillions of microorganisms residing in the human gut form a complex microbial ecosystem, which is deeply intertwined with human biology [34]. Therefore, it is important to view the gut microbiome from an ecological perspective, although it is formidable due to fluctuations over time and variations between individuals [34]. Resilience is the extent of perturbation that an ecosystem can tolerate before it equilibrates toward a different state [92]. Resilience of the gut microbiome is crucial to colonization resistance to pathogens [34,93]. Species richness and evenness is key to the resilience of the gut microbial community. Gut microbiome with species-rich communities is less susceptible to perturbation and stress because different species are specialized to each potentially-limiting resources [94]. Moreover, high species richness enables alternative species with similar functions to fill a niche and maintain the diversity when the original species is compromised [95]. The diversity of the gut microbial ecosystem can be compromised by environmental factors (e.g., antibiotics), which makes it less resilient and more susceptible to pathogen invasion. For instance, antibiotics can induce changes in the gut microbiome and metabolic features that increased its susceptibility to *Clostridium difficile* infection [96]. In addition, a core set of gut microbial species across individuals does not exist. However, a functional core microbiome is shared with similar functional gene profiles [97]. Maintaining the functional core of the gut microbiome is indispensable because normal functioning of the human biology relies in part on the essential functions performed by the gut microbiome. However, exposure to toxic environmental chemicals possibly reduces species richness and diversity of the gut microbiome, leading to potential dysfunction.

### 3.3. Interference in Energy Metabolism

Accumulating evidence suggests that the gut microbiome plays a crucial role in energy metabolism. Humans cannot degrade most plant polysaccharides, which instead, can be utilized by the gut bacteria, producing SCFAs that are important energy substrates [80]. Direct evidence supporting the role of the gut microbiome in energy balance is that germ-free rats have reduced intestinal levels of SFCAs and doubled excretion of calories through urine and feces [98,99]. It is suggested that the capacity for the energy harvest of the gut microbiome is correlated with its microbial composition [100], specifically, the ratio of two major phyla *Firmicutes* and *Bacteroidetes*. Moreover, enriched genes encoding enzymes that are important for the initial steps of complex carbohydrate metabolism were found in the gut microbiome of obese mice [101]. Studies showed that the energy balance and body weight of the host is associated with the gut microbiome types. For instance, germ-free mice with fecal microbiota transplantation from obese mice gained more weight than that from lean mice [100]. Likewise, mice with the microbiota from people afflicted with Kwashiorkor, a form of malnutrition, suffered severe weight loss [102]. Thus, it is possible that gut microbiome toxicity interferes with the energy extraction and harvest, leading to diseases such as obesity or malnutrition.

## 4. Biomarkers and Assessment of Gut Microbiome Toxicity

Routine toxicity screening and evaluation of environmental chemicals fail to consider gut microbiome toxicity. There is no toxic endpoint currently established to report the relative toxic effects of certain chemicals on the gut microbiome. Thus, it is imperative to assess the functional alterations induced by various environmental chemicals, or at least the chemicals of frequent and long-term exposure (e.g., artificial sweeteners). Current approaches for the assessment of gut microbiome toxicity mainly comprise an integration of animal models (e.g., mouse, rat, and germ-free animals) and the meta-omics toolkit [10]. The use of animal models enables us to mimic the progress of gut microbiome toxicity under environmental exposures; the meta-omics toolkit comprises sequencing-based gene profiling and mass spectrometry-based metabolite profiling. Meta-omics comprise approaches that reveal both compositional levels and functional levels. Compositional profiling, that is, taxonomic profiling, provides details of the microbial constitution and diversity. However, knowing the taxonomic information alone does not necessarily lead to an accurate understanding of microbiome functions due to the existence of functional redundancy in the microbiota [34]. In the context of gut microbiome toxicity, the functional changes including the genes, mRNAs, proteins, and metabolites are what we should emphasize. Furthermore, humanized gnotobiotic mice with gut microbiota more similar to that of humans allow for better elucidation of the interactions between human gut microbiome and the environment [103]. The use of germ-free mice and in vitro techniques extends the observational studies to causality [10]. The accurate assessment of gut microbiome toxicity provides knowledge of how gut microbes react to environmental exposures, offering insights into the mechanistic basis of chemical-induced microbial perturbations and diagnostic markers for microbiota-associated diseases.

In order to diagnose gut microbiome toxicity, specific and effective biomarkers are needed. The gut microbiome and its functions will change under various environmental pressure at almost all times, however, not all changes are necessarily adverse and lead to adverse outcomes. Therefore, it is imperative to develop strategies identifying alterations that adversely influence human health. Currently the techniques and approaches used for gut microbiome assessment are usually at the meta-level; thus, the pinpoint of specific bacterial genes or metabolites that can be used to sensitively indicate environmentally induced dysfunction in the gut microbiome is warranted. Additionally, it should be noted that biomarker development may be on a case-by-case basis. Different xenobiotics would induce distinct gut microbiome changes. The elucidation of the role of the gut microbiome in the toxicity of certain exposure is the premise of biomarker development of gut microbiome toxicity.

Biomarkers are commonly used as primary end points in basic and clinical research, connecting environmental exposures to health outcomes [104]. Incorporating gut microbiome toxicity, our understanding of biomarkers should include functional changes in the gut microbiome as critical indicators in progressions from exposure to microbiome-associated diseases [105]. The functional role of the gut microbiome in host metabolism and physiology is largely determined by microbiome metabolic profiles, especially metabolic pathways and products of gut bacteria. Exposure to a range of xenobiotics would lead to perturbation in microbiome profiles, thereby resulting in functional alterations and gut microbiome toxicity. An in-depth look at microbiome changes upon various environmental exposures will provide insights regarding biomarkers of gut microbiome toxicity induced by specific environmental chemicals. While keeping the host in the picture, development and characterization of sensitive and robust biomarkers of gut microbiome toxicity could spur new advances in environment–microbiome interactions and microbiome-related diseases.

Biomarkers of gut microbiome toxicity could be bacterial species, genes, or metabolites, even a combination of several these markers. Signature changes in the gut microbiome upon exposure to certain chemical could be used to indicate an exposure to or the effect of specific xenobiotics, which provides a novel and potentially less invasive method for environmental health monitoring. More importantly, if the underlying mechanisms of chemical toxicity involves perturbation of the gut microbiome with specific functional alterations, then these alterations can also be used as potential biomarkers of environmentally driven health conditions.

Recent studies have documented the functional changes in the gut microbiome upon exposure to diverse xenobiotics (Table 1) [12,13,14,15,47,51,52,61,62,66,67,68,69,70,76,106,107,108,109]. Consistent changes in gut microbial profiles could be potential biomarkers of gut microbiome toxicity associated with specific chemical exposures. Outlining changing patterns and trajectories of microbial composition offers a sketch of biomarkers for gut microbiome toxicity. The *Firmicutes*/*Bacteroidetes* ratio is suggested to be indicative of energy harvesting capacity in the gut microbiome that is associated with host adiposity [100]. Likewise, *Enterobacteriaceae* is associated with gut inflammation [110]. The ratio of *Firmicutes* and *Bacteroidetes* as well as the abundance of *Enterobacteriaceae* in the gut can be readily changed by chemicals such as carbendazim [108] and aspartame [68]. Thus, such taxonomic characteristics can serve as biomarkers of gut microbiome toxicity associated with health outcomes such as inflammation and obesity. Moreover, distinctive changes in functional profiles such as key metabolites and metabolic pathways could serve as more relevant biomarkers because alterations in functional profiles directly influence the host. For example, arsenic exposure perturbed the gut microbial metabolite profiles, especially indole-containing metabolites, isoflavone metabolites, and bile acids [12]. Alterations in these functional metabolites could be a potential new mechanism of arsenic toxicity, and particularly, changes of these metabolites (e.g., bile acids and indole-containing compounds) can be used as biomarkers of arsenic-induced gut microbiome toxicity. Likewise, consumption of artificial sweeteners is associated with increased levels of pro-inflammatory metabolites and genes in the gut microbiome. This may be used as bioindicators of artificial sweeteners-induced gut microbiome toxicity that consequently leads to inflammation [15,67]. In addition, diazinon changed the bacterial pathways and metabolites involved in neurotransmitters in a gender-dependent manner, indicating that those bacteria-derived neurotransmitters can be biomarkers to probe gut microbiome toxicity arising from chemicals that have neurological toxicity [13]. The gender-dependent effect also indicates individual variation in biomarkers of gut microbiome toxicity resulting from gender differences in the gut microbiome.

More efforts should be put into the search and validation of biomarkers of gut microbiome toxicity, which would further elucidate the link between environmental chemicals and microbiome-related disease. Delineating these microbial changes and elucidating their biological effects is undoubtedly challenging due to the complexities within the gut microbiome as well as the intertwinement between the gut microbiome and other systems including immune, endocrine, and nervous systems. However, recent advances and emerging approaches are enabling progress toward a better understanding of gut microbiome toxicity biomarkers, which will inform toxicology risk assessment and development of therapeutic interventions via modulation of the gut microbiome.

## 5. Gut Microbiome Modulation

It is increasingly acknowledged that one of the crucial mechanisms underlying chemical toxicity is perturbation of the gut microbiome functions. The inclusion of the modulation section corresponds to ‘treatment’ in traditional organ toxicity and related diseases. Therefore, it is reasonable to include the treatment of gut microbiome-associated diseases—gut microbiome modulation.

The gut microbiome is becoming an attractive therapeutic target, especially now with its role well recognized in human health and disease. Current approaches for gut microbiome modulation including fecal microbiota transplantation (FMT), probiotics, and prebiotics are mainly untargeted without predictable outcomes [10]. To move from untargeted toward targeted modulation, a healthy gut microbiome needs to be defined. A consensus on the healthy endpoints of gut microbiome modulation remains elusive, which is a major challenge [111]. Nevertheless, the potential of targeted, hypothesis-driven gut microbiome modulation has been demonstrated in some recent studies. Use of whole foods or food components as dietary intervention to modulate the gut microbiome has received increasing attention due to their low toxicity profiles and high patient compliance [112]. Even with well acknowledged health benefits and capacity of gut microbiome modulation, it should be noted that there is evidence that dietary fiber could also possibly exacerbate gut conditions [113,114]. Here, we use *Akkermansia muciniphila* (*A. muciniphila*) as an example to review recent progress on attempts at targeted microbiome modulation.

*A. muciniphila*, a mucin-degrading bacterium commonly present in human and mouse gut microbiome, has many probiotic effects in gut barrier function, glucose homeostasis, and inflammation in humans and diverse animal models [115,116,117,118,119]. Several studies reported targeted gut microbiome modulation with increased *A. muciniphila* population via consumption of whole foods or food components. For example, consumption of several berry fruits including cranberries and raspberries promoted increased content and enhanced function of *A. muciniphila* in the gut microbiome in rodent studies. Specifically, cranberry extract improved insulin sensitivity and reduced weight gain in concert with a significant increase of *A. muciniphila* in diet-induced obese mice [120]. Likewise, black raspberries boosted *A. muciniphila* population in the gut microbiome together with profound changes in microbial functions and metabolites [121,122,123]. The polyphenols abundant in berry fruits could be a reason that *A. muciniphila* thrives. Feeding polyphenols from grapes to mice showed similar results with a drastic increase of *A. muciniphila* [124]. The gut microbiome offers a link between polyphenols and their diverse beneficial effects because polyphenols are poorly absorbed and metabolized by the human body [124]. Meanwhile, *A. muciniphila* uses mucin as carbon, nitrogen, and energy sources [125]. Goblet cells are the major producer of mucin in the intestinal epithelium [126]. It is reported that the number of goblet cells and the thickness of intestinal mucosa were increased in rats fed oligofructose [127]. Therefore, oligofructose may be an alternative factor for the increase of *A. muciniphila* in mice fed berries, which is supported by the study that administration of oligofructose did increase the *A. muciniphila* population in the gut microbiome of mice [128]. Of particular interest, metformin, medication to treat type 2 diabetes, also promotes *A. muciniphila* population in the gut microbiome, which is believed to contribute to its therapeutic effects [129,130]. Perturbation by environmental toxic chemicals and modulation by dietary components regarding the gut microbiome are fundamentally similar, except with different expected outcomes. Knowledge of how gut microbes react to xenobiotics and dietary components will address gaps in our understanding of both perturbation and modulation of the gut microbiome.

## 6. Conclusions

**Figure 2:**
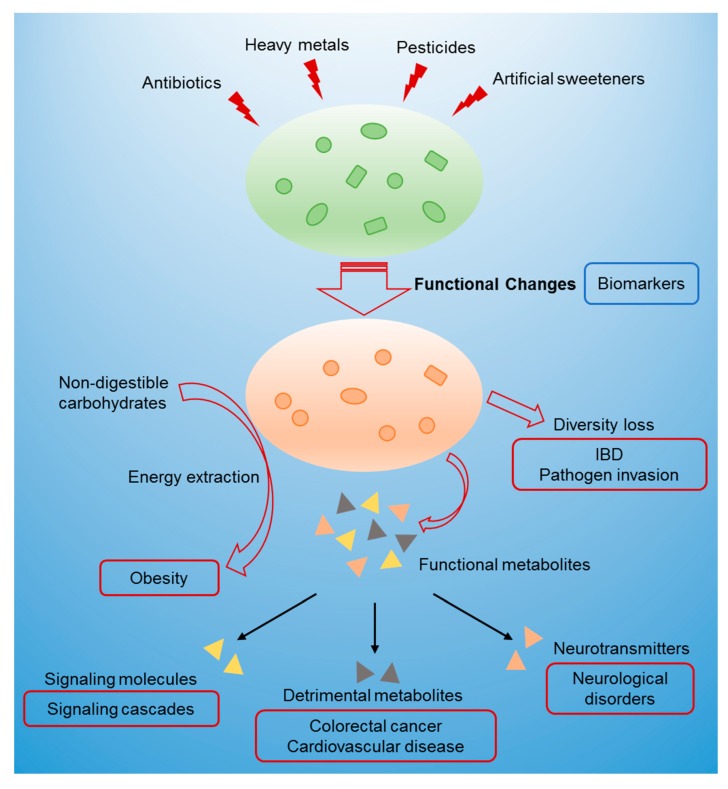
Schematic representation of how gut microbiome toxicity connects the environment and microbiota-associated diseases. Triangles of different colors at the bottom represent functional metabolites produced by a perturbed gut microbiome such as signaling molecules, detrimental metabolites, and neurotransmitters, which could potentially contribute to adverse health outcomes.

To summarize, exposure to xenobiotics such as antibiotics, heavy metals, and artificial sweeteners induces gut microbiome toxicity. Compositional alterations and functional changes occur along with this process in the gut microbiome, which can serve as potential biomarkers of gut microbiome toxicity. These chemical-induced perturbations lead to human diseases via several mechanisms including changes in the metabolite profiles, diversity loss, and altered energy metabolism (Figure 2).

Given the continued enthusiasm in gut microbiome research, it is now an opportune time to examine environmentally induced gut microbiome alterations through the lens of toxicology. Although strong connection has already been established between gut microbiome disturbances and environmental exposure, the mechanisms of these disturbances and health implications await future studies. The goal of this paper was to establish and emphasize gut microbiome toxicity with the definition of chemical-driven functional damage in the gut microbiome and to review the current state of knowledge regarding biomarker, assessment, and modulation of gut microbiome toxicity. Toxic effects of various environmental agents on the gut microbiome must not be underappreciated. The integration of gut microbiome toxicity endpoints into the evaluation of chemical toxicity will provide a better understanding of the associations between the environment and human health and disease, and will facilitate the development of diagnostic markers and therapeutic interventions.

## Figures and Tables

**Table 1:** Microbiome changes associated with specific chemical exposure that can serve as potential biomarkers of gut microbiome toxicity.

Class	Chemical	Potential Biomarker of Gut Microbiome Toxicity			Adverse Outcome	Reference
		Gut Bacteria	Gene and Pathway	Metabolite		
Heavy metal	Arsenic	*Catabacteriaceae* ↓ *Clostridiaceae* ↓ *Clostridiales Family XIII Incertae Sedis* ↑ *Erysipelotrichaceae* ↓	LPS, DNA repair, multi-drug resistance ↑	Indolelatic acid, Indole-3-carbinol, daidzein, glycocholic acid↓		[12,47]
	Cadmium	*γ-Proteobacteria* ↓ *Firmicutes/Bacteroidetes ratio* ↓		LPS ↑	Inflammation	[106]
	Lead	*Ruminococcus* ↓ *Oscillospira* ↓	Oxidative stress ↑	Vitamin E and bile acids ↓		[52]
	Manganese	*Firmicutes*↑ *Bacterodetes* ↓ in males *Firmicutes* ↓ in females	Phenylalanine synthesis ↑ in females, phenylalanine synthesis ↓ in males	Vitamin E↓, phenylalanine ↑ in females		[51]
Pesticide	Carbamate aldicarb	*Clostridium* ↑ *Anaerostipes* ↓	Virulence, adhesion and bacteriocins ↑	1-Methylnicotinamide ↑		[109]
	Carbendazim	*Firmicutes*/*Bacteroidetes* ratio ↑			Inflammation	[108]
	Diazinon	*Lachnospiraceae Johnsonella* ↑ in females, *Lachnospiraceae Johnsonella* ↓ in males	Tryptophase ↑ in males	Taurine and glycine ↓ in males		[13]
	Imazalil	*Clostridiales* ↑ *Lachnospiraceae* ↑			Inflammation	[107]
	Malathion	*Mogibacteriaceae* ↑	Quorum sensing, virulence and pathogenicity ↑			[62]
Artificial sweetener	Ace-K	*Bacteroides*, *Anaerostipes* and *Sutterella* ↑ in males	Carbohydrate metabolism ↑ in males, LPS ↑	Pyruvic acid ↑ in males	Obesity in males	[61]
	Aspartame	*Enterobacteriaceae* ↑ *Clostridium leptum* ↑ *Firmicutes*/*Bacteroidetes* ratio ↓		Propionate ↑	Diabetes	[68]
	Neotame	*Firmicutes*/*Bacteroidetes* ratio ↓	Butyrate biosynthesis ↓	Malic acid and glyceric acid ↓ cholesterol ↑		[69]
	Saccharin	*Bacteroides* ↑ *Clostridiales* ↑	Glycan degradation ↑	Acetate and propionate ↑	Diabetes	[66]
	Saccharin	*Corynebacterium* ↑ *Roseburia* ↑ *Ruminococcus* ↓ *Turicibacter* ↑	LPS biosynthesis, fimbrial proteins and bacterial toxins ↑	Equol ↓, quinolinic acid ↑	Inflammation	[15]
	Sucralose	*Christensenellaceae* ↑ *Clostridiaceae* ↑ *Erysipelotrichaceae* ↓	LPS biosynthesis, fimbrial proteins and bacterial toxins ↑	Quorum sensing molecules ↓, bile acids ↓	Inflammation	[67]
Other	Emulsifiers (P80 and CMC)	*Akkermansia*, *Proteobacteria* ↑		LPS and flagellin ↑	Colitis	[70]
	Nicotine	*F16*↓ in females, *F16* ↑ in males	Oxidative stress and DNA repair ↑ in male	Serine and glycine ↑ in females, serine and glycine ↓ in males		[76]
	Triclosan	*Turicibacteraceae* ↓ *Clostridiaceae* ↓ *Lactobacillaceae* ↑ *Streptococcaceae* ↑ *Christensenellaceae*↓	Antibiotic resistance and metal resistance ↑ Stress response ↑			[14]
